# Agreement of the morphometric dimensions determined by traditional and image analysis methods for Arabian and Thoroughbred horses

**DOI:** 10.5194/aab-68-253-2025

**Published:** 2025-04-02

**Authors:** Necmettin Ünal, Yavuzkan Paksoy, Ömer Faruk Güngör

**Affiliations:** 1 Department of Animal Breeding and Husbandry, Ankara University Faculty of Veterinary Medicine, Ankara 06110, Türkiye; 2 Necmettin Erbakan University, Eregli Kemal Akman Vocational School, Department of Plant and Animal Production, Konya 42090, Türkiye; 3 Department of Veterinary, Vocational School, Bolu Abant Izzet Baysal University, Mudurnu, Bolu 14800, Türkiye

## Abstract

The aim of this method agreement study was to agree on traditional and digital measuring methods using a smartphone. For this purpose, in total, 48 purebred horses (24 Arabian and 24 Thoroughbred) were used in this study. Lengths of the head, neck, body, back, and rump; heights of the shoulder, withers, rump, and toe; widths of the head, chest, and front chest; and depths of the chest dimensions were taken from the horses to evaluate the agreement of the traditional and digital measuring method results. The results of these two methods were compared by linear regression and Bland–Altman plot analysis. The results of the digital analysis were strongly correlated with the traditional method results for Arabian and Thoroughbred horses ( r=1.000 , P<0.001 ). As a result of the Bland–Altman plot analysis, 95.2 % of the differentiation value points of Arabian horses and 94.9 % of the differentiation value points of Thoroughbred horses were within the 95 % limit-of-agreement (LOA) interval. The values of agreement indices (AIs) for Arabian horses were between 0.92 and 1.00, and the values for Thoroughbred horses were between 0.95 and 0.99. These results indicate great agreement between these two methods. The main limitations of this study are the study population and measurement regions, which are limited to defined regions. This study concludes that, although all dimensions have not been examined, the length, width, height, and depth dimensions of these horses demonstrate excellent accuracy between traditional and digital assessment methods.

## 1 Introduction

Since technology has advanced and horses have lost some of their historical significance, they are largely used as sporting animals (Akçapınar and Özbeyaz, 1999; Özbeyaz et al., 2016) The most popular breeds of horses worldwide, Arabian and Thoroughbred horses, are typically raised for racing these days. The Arabian horse, which is one of the warm-blooded horse breeds, is known for its durability, performance, and good proportion of body measurements. Thoroughbred horses, also one of the warm-blooded horse breeds, are known for their excellent speed (Akçapınar and Özbeyaz, 1999; Hacan et al., 2024).

The different breeds have different morphometric traits, and this information is necessary for official control and commerce of the horses in order to determine the breed characteristics. In addition, morphometric traits are a good indicator for the evaluation of body development and the determination of breeding stocks. Horse speed and jumping abilities are commonly used to assess horse performance. The harmony of the body parts generally appears to indicate a factor that directly affects the speed and jumping ability of equestrian horses. The most popular tools for measuring morphometric traits are goniometers and measuring tapes or sticks (Freitag et al., 2021; José Sánchez et al., 2013). For the purpose of genetic improvement in horse breeding, it is crucial to measure and assess the fundamental body dimensions of horses, including the height of the fetlock, height of the rump, depth of the chest, and height of the withers. Horse welfare is guaranteed and risk is decreased by replacing human measurements with technological tools like mobile phones and cameras (Ferreira Padilha et al., 2017; Matsuura et al., 2021). One of the primary selection factors in horse breeding, along with performance and health, is conformation. Specifically, young horses' conformation evaluation enables performance selection prior to their participation in equestrian events (Gmel et al., 2018; Reich et al., 2024). According to recent studies, image analyses are more beneficial for measuring body parts with a camera because they have a high accuracy rate and are simple to assess when using digital photographs (Freitag et al., 2021; Mariz et al., 2015; White et al., 2008). Thus, during the past few decades, much research has been conducted on determining morphometric traits using computer-aided measurement with photographs or recordings (Freitag et al., 2021; Genç, 2018; Hunt et al., 1999; dos Santos et al., 2017; White et al., 2008). According to the majority of these studies, the most effective results may be obtained by adjusting the steady animal body, recognizing reference points in this location, and using appropriate camera angles. Nowadays, the development of smartphone technology and its program software makes it possible to easily measure morphometric traits using smartphones.

In this study, the measurements identified by traditional methods and digital methods using smartphones were compared for consistency. For this reason, some horizontal and vertical body measurements commonly determined for Arabian and Thoroughbred horses at various ages were obtained from the front angle using both digital and traditional methods, and the degree of agreement between the two methods was evaluated.

## 2 Materials and methods

### 2.1 Animals

The animal materials used in this study consisted of 24 Thoroughbred and 24 Arabian equestrian horses, ranging in age from 4 to 16 years. These horses were chosen randomly in 2023 from private farms in the Turkish provinces of Adana (37°06^′^15.2^′′^ N, 35°21^′^07.3^′′^ E; 37°01^′^43.7^′′^ N, 35°20^′^34.0^′′^ E; 36°58^′^49.8^′′^ N, 35°10^′^46.5^′′^ E; 37°07^′^06.7^′′^ N, 35°30^′^56.6^′′^ E; 37°04^′^35.5^′′^ N, 35°22^′^38.4^′′^ E), Mersin (36°50^′^07.7^′′^ N, 34°25^′^05.2^′′^ E; 36°44^′^03.0^′′^ N, 34°31^′^07.3^′′^ E), Osmaniye (37°08^′^25.6^′′^ N, 36°21^′^12.2^′′^ E), Konya (37°31^′^08.1^′′^ N, 34°05^′^19.5^′′^ E), Niğde (37°56^′^44.8^′′^ N, 34°39^′^06.0^′′^ E), and Kayseri (38°38^′^23.6^′′^ N, 35°29^′^54.5^′′^ E).

### 2.2 Measurements

**Figure 1:**
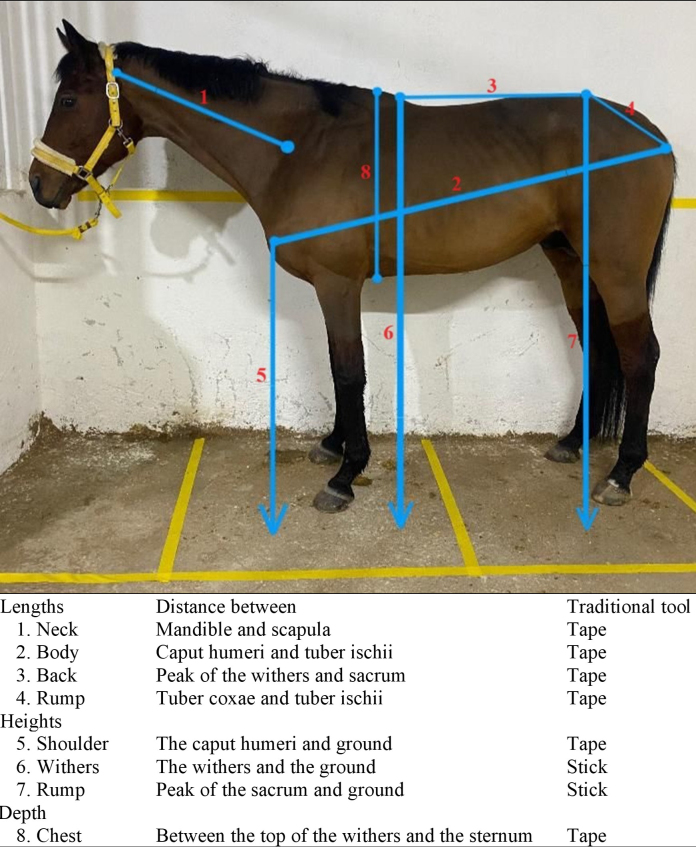
Common dimension regions and definitions.

**Figure 2:**
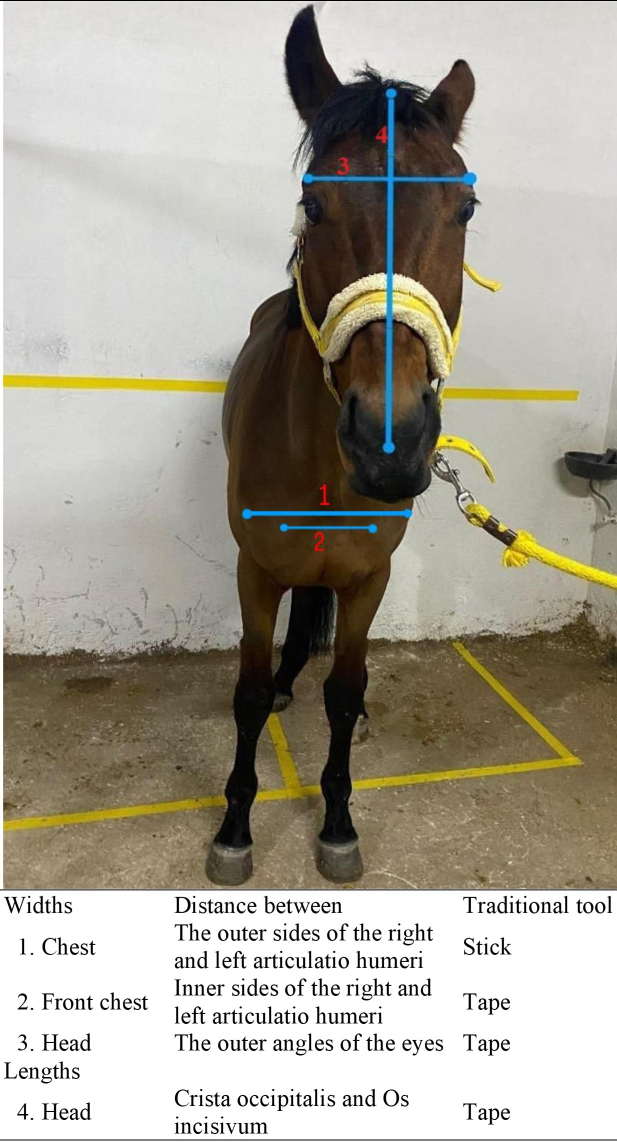
Common dimension regions and definitions.

**Figure 3:**
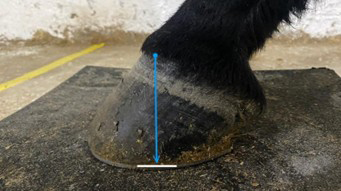
Toe height (the vertical line from the white line to the horseshoe).

These dimensions of regions (defined in Figs. 1, 2, and 3) were first measured using a traditional measuring tape or stick, and then they were measured again using a smartphone-based device (the iPhone 11 with a 12 MP dual camera, f/1.8 wide angle, and f/2.4 ultra-wide aperture). Using measurement applications for digital analysis is only compatible with this device. With this application, all body measurements were obtained manually while the horses were standing in their typical poses on level ground and in front of a level wall (IPhone, 2023).

The digital measurements shown in Fig. 1 were taken from the left side at 3 m in length and 1 m in height. The digital measurements in Fig. 2 were taken from the front side at 2 m in length and 1 m in height. The digital measurements in Fig. 3 of the toe height were taken from the left side at 1 m in length and 20 cm in height, with the toe stabilized on a surface 2 cm high. The horses were not given anaesthetic drugs because it was believed that some measured values might be lower due to the anaesthetic medicines' tendency to relax the muscles.

### 2.3 Data analysis

The Bland–Altman plot analysis was primarily used to determine the difference between the two values determined by traditional and digital measuring methods, but linear regression analysis was first conducted to illustrate the limit of the relationship between the traditional method and the image analysis findings.

Bland–Altman plot graphics were made of Arabian and Thoroughbred horses. In the first step for the Bland–Altman plot, one sample T test was performed to determine the mean bias and its standard deviation. The means [ Da+Db)/2 ] of values determined by the traditional method (Da) and image analysis (Db) are presented on the x axis, and the differences (Da - Db) between the traditional method and the image analysis results are presented on the y axis. The confidence intervals of the differences were calculated according to the 95 % limit-of-agreement (LOA) values as follows (Bland and Altman, 1986):

1upper 95 % LOA=mean of differences+1.96×standard deviation,2lower 95 % LOA=mean of differences-1.96×standard deviation.

In the second step for the Bland–Altman plot, linear regression analysis was performed to determine the proportional bias using the differences (dependent value) and the means (independent value) (Ludbrook, 2010).

In the last step, the upper and lower 95 % LOA values and the agreement indices (AIs) between traditional and image measurements were calculated for Arabian and Thoroughbred horses for each measurement region. AIs were calculated as follows (Bland and Altman, 1986, 2003; Filippi et al., 1995; van der Vlugt-Meijer et al., 2006):

3 $$\mathrm{AI}=1-\frac{\left|\mathrm{Da}-\text{Db}\right|}{\left(\mathrm{Da}+\text{Db}\right)/2}.$$

The SPSS Version 22 software was used for all of the statistical analyses and illustrations (SPSS, 2013).

## 3 Results

### 3.1 Linear regressions

**Figure 4:**
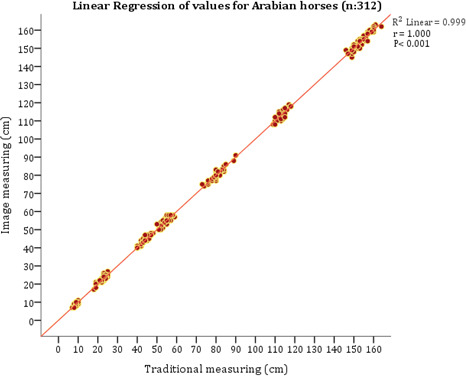
Linear regression between the image and traditional measuring results obtained from Arabian horses (image measuring mean = 75.76 cm, traditional measuring mean = 75.65 cm, and y=0.230+0.998⋅x ).

**Figure 5:**
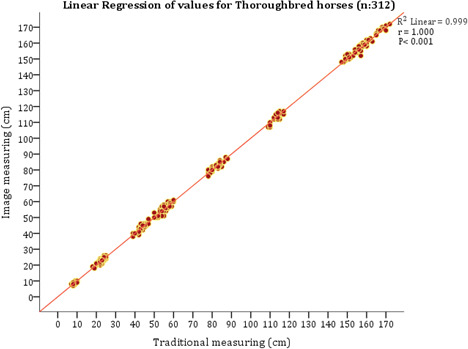
Linear regression between the image and traditional measuring results obtained from Thoroughbred horses (image measuring mean = 77.15 cm, traditional measuring mean = 77.02 cm, and y=-0.044+1.002⋅x ).

The linear regression of paired values for Arabian and Thoroughbred horses (Figs. 4 and 5) obtained using the image and traditional methods shows a highly significant ( P<0.001 ) correlation ( r=1.000 ).

### 3.2 Bland–Altman scatterplots

**Figure 6:**
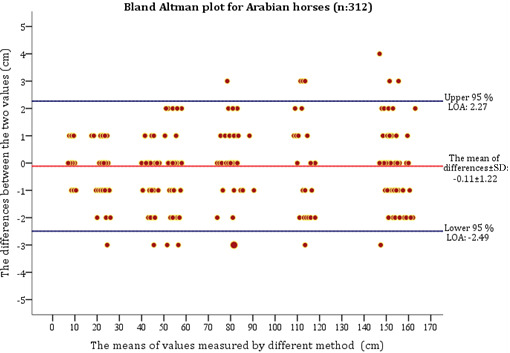
Bland–Altman plot of the Arabian horse results. The one-sample t test ( t=-1.63 , P<0.104 ) and linear regression ( $R^{2}=0.003$ , r=0.052 , B value of the non-standardized coefficient = 0.001, P<0.357 ) are defined. Overlapping binary values out of LOA are presented as larger symbols than the others.

**Figure 7:**
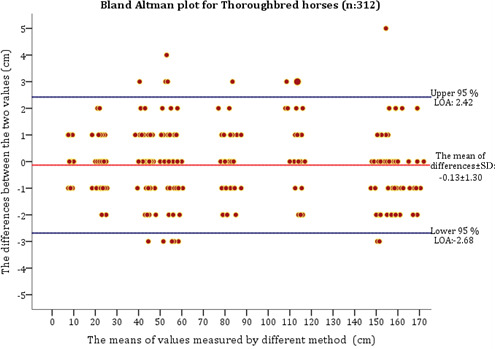
Bland–Altman plot of the Thoroughbred horse results. The one-sample t test ( t=-1.78 , P<0.076 ) and linear regression ( $R^{2}=0.011$ , r=0.103 , B value of the non-standardized coefficient = -0.003 , P<0.070 ) are defined. Overlapping binary values out of LOA are presented as larger symbols than the others.

For Arabian horses, the intervals of the upper and lower 95 % LOA were between 2.27 and -2.49 cm, and 15 values were located outside these intervals (Fig. 6). The intervals of the upper and lower 95 % LOA for the Thoroughbred horses were between 2.42 and -2.68 cm, and 16 values, 7 of which were negative and 9 positive, were located outside these intervals (Fig. 7).

**Figure 8:**
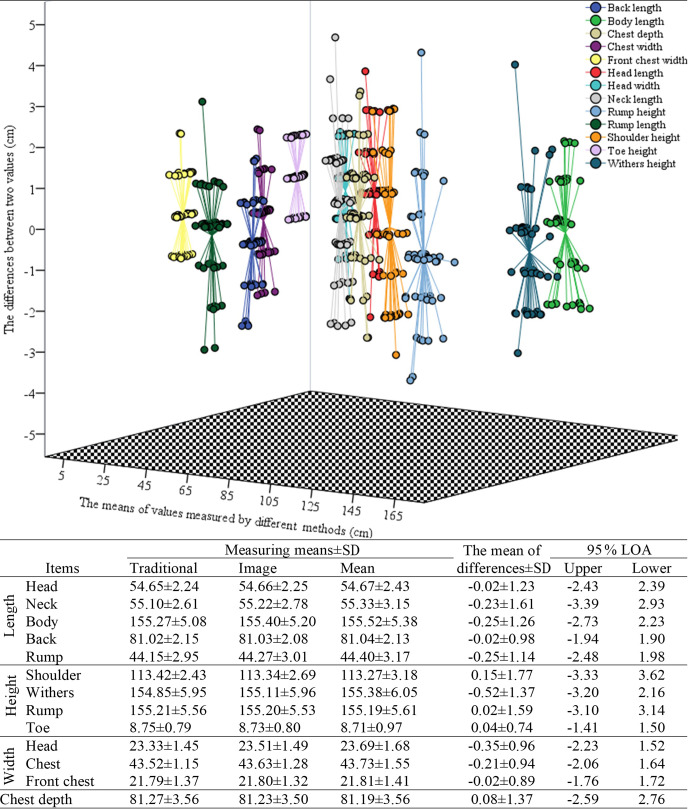
Bland–Altman scatterplot of all the measurements according to the regions ( n = 48 for each item).

For the measurement region results, the Bland–Altman scatterplot is given with the measurement means, the mean of the differences, and the intervals of the upper and lower 95 % LOA in Fig. 8.

### 3.3 Agreement indices of the measured regions

AI values were between 0.92 and 1.00 for the Arabian horses (Table 1). The upper and lower 95 % LOA results for these horses were between 1.37 and 3.56 cm and between -3.56 and -1.63 cm, respectively. For the Thoroughbred horses, the AI values were determined to be between 0.95 and 0.99. The minimum and maximum values of 95 % LOA were 1.29 and 3.75 cm for the upper 95 % and -3.75 and -1.20 cm for the lower 95 % (Table 2).

**Table 1:** Measurement results, AIs, and 95 % LOA for Arabian horses ( n = 24 for each item).

Items	Measuring means ± SD			Agreement	95 % LOA	
	Traditional	Image	Mean	Index ± SD	Upper	Lower
Lengths						
Head	54.50±2.04	54.75±1.94	54.63±1.93	0.99±0.02	1.69	-2.19
Neck	54.67±2.48	54.88±2.97	54.77±2.63	0.98±0.02	2.74	-3.15
Body	153.67±3.47	153.79±3.99	153.73±3.71	1.00±0.00	1.82	-2.07
Back	80.83±2.39	80.71±2.35	80.77±2.33	0.99±0.01	1.89	-1.64
Rump	43.92±2.57	44.33±2.71	44.13±2.59	0.99±0.02	1.58	-2.41
Heights						
Shoulder	113.17±2.68	113.17±3.40	113.17±2.92	0.99±0.01	3.56	-3.56
Withers	151.79±3.46	151.88±3.66	151.83±3.50	0.99±0.01	2.62	-2.79
Rump	153.08±4.05	152.96±4.36	153.02±4.13	0.99±0.01	3.25	-3.00
Toe	8.79±0.78	8.79±1.02	8.79±0.81	0.92±0.05	1.63	-1.63
Widths						
Head	23.13±1.73	23.54±2.11	23.33±1.85	0.96±0.03	1.74	-2.57
Chest	43.29±1.04	43.63±1.53	43.46±1.23	0.98±0.01	1.37	-2.03
Front chest	21.83±1.31	21.83±1.31	21.83±1.24	0.97±0.02	1.63	-1.63
Depth						
Chest	80.75±4.11	80.63±4.17	80.69±4.07	0.99±0.01	3.09	-2.84

LOA: limit of agreement. SD: standard deviation. AIs: agreement indices.

**Table 2:** Measurement results, AIs, and 95 % LOA for Thoroughbred horses ( n = 24 for each item).

Items	Measuring means ± SD			Agreement	95 % LOA	
	Traditional	Image	Mean	Index ± SD	Upper	Lower
Lengths						
Head	54.79±2.45	54.58±2.87	54.69±2.57	0.98±0.02	2.98	-2.56
Neck	55.54±2.72	55.79±3.32	55.67±2.91	0.98±0.02	3.18	-3.68
Body	156.88±5.94	157.25±6.09	157.06±5.97	0.99±0.01	2.56	-3.31
Back	81.21±1.91	81.38±1.88	81.29±1.82	0.99±0.01	1.89	-2.22
Rump	44.38±3.32	44.46±3.64	44.42±3.43	0.98±0.02	2.36	-2.53
Heights						
Shoulder	113.67±2.18	113.38±3.02	113.52±2.48	0.99±0.01	3.73	-3.15
Withers	157.92±6.38	158.88±5.98	158.40±6.15	0.99±0.01	1.46	-3.37
Rump	157.33±6.11	157.33±6.00	157.33±5.98	0.99±0.01	3.75	-3.75
Toe	8.71±0.81	8.63±0.92	8.67±0.80	0.95±0.06	1.36	-1.20
Widths						
Head	23.54±1.10	23.83±1.13	23.69±1.04	0.98±0.03	1.29	-1.87
Chest	43.75±1.22	43.83±1.61	43.79±1.33	0.98±0.02	1.91	-2.08
Front chest	21.75±1.45	21.79±1.53	21.77±1.41	0.97±0.03	1.83	-1.91
Depth						
Chest	81.79±2.90	81.75±2.82	81.77±2.79	0.99±0.01	2.46	-2.37

LOA: limit of agreement. SD: standard deviation. AIs: agreement indices.

## 4 Discussion

For horses, body measurements and the proportion of these values reflect breed characteristics, orthopaedic health, and physical performance (Paksoy and Ünal, 2019; Senna et al., 2015). The determination of these values could be quite difficult for the evaluator and stressful for horses with harsh temperaments. Additionally, it is stated that each measurement takes roughly 1 min, which is a very long period when numerous measures are taken (dos Santos et al., 2017). Because of the potential for measurement mistakes caused by these undesired situations, image measurement is a better method than traditional methods. Moreover, image analysis is faster and more secure than traditional techniques.

### 4.1 Linear regression of the two methods

For the information in Figs. 4 and 5, $R^{2}=0.999$ and r=1.000 ( P<0.001 ), the points in the linearly regressing scatterplot accumulated on the linear regression line like tight clouds for the two breeds. The results of the traditional method correlated perfectly with the image measurement values, as implied by this more significant correlation. These findings are consistent with other research findings (Freitag et al., 2021; dos Santos et al., 2017), which is a good sign for deducing the efficacy of digital measurement, even though they do not show method agreement because the correlation evaluates a consistent proportion of measurement pairs, not similarity of measurement value pairs (Bland and Altman, 1986).

### 4.2 Bland–Altman scatterplot of Arabian and Thoroughbred horses

The differentiation values of the two methods for the Arabian and Thoroughbred horses (Figs. 6 and 7) have shown insignificant ( t=-1.63 , P<0.104 ; t=-1.78 , P<0.076 ) mean biases (mean differences: Arabian horses = -0.11 cm and Thoroughbred horses = -0.13 cm). These lower mean difference values and insignificant mean biases, though not enough, are signs of acceptable agreement between the two methods, and this insignificant mean bias is necessary for continuing Bland–Altman analysis (Bland and Altman, 1986; Ludbrook, 2010). The upper and lower 95 % LOA values for Arabian horses were between 2.27 and -2.49 cm and 95.2 % of the differentiation value points were within this 95 % LOA interval, with only 4.8 % of the differentiation value points outside this range. For Thoroughbred horses, these results were 2.42 and -2.68 and 94.9 % of the differentiation value points were within this 95 % LOA interval, with only 5.1 % of the differentiation value points outside this range. Bland and Altman (2003) reported that approximately 95 % of the differentiation value points should be within the 95 % LOA limits. In this context, the scattering results of differentiation value points for the two breeds in this study can be evaluated as good indicators of the agreement between the two methods.

For coherence between the two methods, the dispersions of the differentiation points in the scatterplot are expected to be close to the main difference line and unaffected by the size of the mean value (Doğan, 2018; Ludbrook, 2010). In this study, the differences between the value pairs obtained by the different methods did not change as the mean values increased. In other words, the scatterplot of the differentiation values against the mean of these two-value pairs in Figs. 6 and 7 is partially like a rectangle shape. In addition, the differentiation value point dispersions in Figs. 6 and 7 were generally too closely scattered around the mean difference lines. These two results verify the agreement between these two methods. These results were also consistent with the results of the region-specific Bland–Altman plot (Fig. 8). Owing to the fact that the Bland–Altman scatterplot and the LOA values of the measurement regions were evaluated, the cluster of the region measurement means in the scatterplot was generally uniform, and the upper and lower 95 % LOA values of the region measurements were generally between 3 and -3 cm. The clusters of neck length, withers height, and rump height were a bit spread out because of their wider LOA ranges; therefore, this was the expected result. Additionally, when the region measurement means of the image and traditional method analyses are compared, almost the same results were obtained for each region (Fig. 8).

Ludbrook (2010) suggested that the B (beta) value of the non-standardized coefficient (this value represents the slope of the line between the predictor variable and the dependent variable) should be closer to zero and that the regression result between the differences of measuring pairs (the dependent value) and the means of measuring pairs (the independent value) should be insignificant for applying Bland–Altman analysis because these results infer no proportional bias. In this study, there were no proportional biases for the two breeds because the B values and regression results of Arabian ( B value = 0.001, P<0.357 ) and Thoroughbred ( B value = -0.003 , P<0.070 ) horses were obtained in accordance with this statement.

### 4.3 Agreement indices of the measured regions of Arabian and Thoroughbred horses

When the mean values of the image and traditional method analyses are compared, almost the same results are understood to be obtained for each region of Arabian and Thoroughbred horses (Tables 1 and 2), and these results obtained in both breeds and in all regions are a positive result for the agreement of the two methods.

AIs ≥0.90 are reported to be a high-agreement result between the two methods (van der Vlugt-Meijer et al., 2006; White et al., 2008). In this study, the AI values were between 0.92 and 1.00 for Arabian horses and between 0.95 and 0.99 for Thoroughbred horses. These outcomes could be seen as a good match between the two methods. When all the AI values were evaluated, they were found to be higher than 0.98, except for head widths (0.96), front chest widths (0.97), and toe heights (0.92) for Arabian horses and front chest widths (0.97) and toe heights (0.95) for Thoroughbred horses. The common thread in these AI values, which are lower than those of the others, is that they have lower mean measurement values than those of the others. According to these results, it could be said that careful measurement should be performed in low-value dimensions.

## 5 Conclusion

The comparisons of the traditional method and image analysis pair and their statistical results were evaluated, and strong agreement was identified in this study. In conclusion, the study's findings demonstrated that, with careful measurement of Arabian and Thoroughbred horses, the dimensions of length, width, height, and depth found in the study could be accurately ascertained by digital analysis utilizing a smartphone. The use of this method on the farms for these measurements is clearly easier and more convenient, especially for horses with harsh temperaments.

## Data Availability

The datasets presented in this study are available from the corresponding author upon request.
